# Knowledge and practices regarding malaria and the National Treatment Guidelines among public health workers in Equatorial Guinea

**DOI:** 10.1186/s12936-020-03528-7

**Published:** 2021-01-07

**Authors:** Marta Blanco, Pablo Suárez-Sanchez, Belén García, Jesús Nzang, Policarpo Ncogo, Matilde Riloha, Pedro Berzosa, Agustín Benito, María Romay-Barja

**Affiliations:** 1 Centro Nacional de Medicina Tropical, Instituto de Salud Carlos III, Madrid, Spain; 2 Servicio de Medicina Preventiva, Hospital Virgen de la Salud, Toledo, Spain; 3 Servicio de Medicina Preventiva, Hospital Clínico San Carlos, Madrid, Spain; 4 Fundación Estatal, Salud, Infancia y Bienestar Social (FCSAI), Madrid, Spain; 5 Ministerio de Salud y Bienestar Social, Malabo, Equatorial Guinea; 6 Red de Investigación Colaborativa en Enfermedades Tropicales, RICET, Madrid, Spain

**Keywords:** Malaria, Treatment, Protocols, Practitioners, Behavior

## Abstract

**Background:**

In 2018, an estimated 228 million cases of malaria occurred worldwide. Countries are far from having achieved reasonable levels of national protocol compliance among health workers. Lack of awareness of treatment protocols and treatment resistance by prescribers threatens to undermine progress when it comes to reducing the prevalence of this disease. This study sought to evaluate the degree of knowledge and practices regarding malaria diagnosis and treatment amongst prescribers working at the public health facilities of Bata, Equatorial Guinea.

**Methods:**

A cross-sectional survey was conducted in October-December 2017 amongst all public health professionals who attended patients under the age of 15 years, with suspected malaria in the Bata District of Equatorial Guinea. Practitioners were asked about their practices and knowledge of malaria and the National Malaria Treatment Guidelines. A bivariate analysis and a logistic regression model were used to determine factors associated with their knowledge.

**Results:**

Among the 44 practitioners interviewed, 59.1% worked at a Health Centre and 40.9% at the District Hospital of Bata. Important differences in knowledge and practices between hospital and health centre workers were found. Clinical diagnosis was more frequently by practitioners at the health centres (*p* = 0.059), while microscopy confirmation was more frequent at regional hospital (100%). Intramuscular artemether was the anti-malarial most administrated at the health centres (50.0%), while artemether-lumefantrine was the treatment most used at the regional hospital (66.7%). Most practitioners working at public health facilities (63.6%) have a low level of knowledge regarding the National Malaria Treatment Guidelines. While knowledge regarding malaria, the National Malaria Treatment Guidelines and treatment resistances is low, it was higher amongst hospital workers than amongst practitioners at health centres.

**Conclusions:**

It is essential to reinforce practitioners’ knowledge, treatment and diagnosis practices and use of the National Malaria Treatment Guidelines in order to improve malaria case management and disease control in the region. A specific malaria training programme ensuring ongoing updates training is necessary in order to ensure that greater experience does not entail obsolete knowledge and, consequently, inadequate diagnosis and treatment practices.

## Background

In 2018, an estimated 228 million cases of malaria occurred worldwide. Fifteen countries in sub-Saharan Africa carry almost 80% of the global malaria mortality [1]. Children under five are the most vulnerable group affected by malaria [2]. In 2018, they accounted for 67% of all malaria deaths worldwide [1]. Malaria also has an important impact on health expenditure, days of work lost and school absenteeism [3].

Disease diagnosis and early treatment are of prime importance in order to reduce the rates of morbidity and mortality. Treating malaria-infected patients within 24–48 h after fever onset is likely to prevent further transmission of the parasite [4]. It is critical that populations living in malaria-endemic areas should have access to reliable diagnostics and effective treatment to control de disease.

Malaria is endemic in Equatorial Guinea with stable transmission, and it continues to be a major cause of morbidity and mortality among children under 5 years of age. The prevalence of malaria in 2011 in children under five was 13% in the Insular Region and 59% in the Continental Region [5].

National programmes for malaria control must adapt the World Health Organization (WHO) recommendations to the local context regarding the use of rapid diagnostic tests (RDT) or microscopy to confirm suspected malaria cases and the prescription of the most appropriate artemisinin-based combination therapy (ACT). ACT is considered a highly effective and well tolerated treatment, one that has contributed substantially to reducing global morbidity and mortality in the case of malaria [6]. However, the continued use of oral artemisinin-based monotherapy is considered to be one of the main reasons for the development and spread of resistance to artemisinin and its derivatives [7]. Unfortunately, countries are far from having achieved reasonable levels of national protocol compliance among health workers [8]. Lack of awareness of treatment protocols and treatment resistances by prescribers threatens to undermine progress when it comes to reducing the prevalence of this disease [9].

Health workers’ knowledge and practices regarding malaria diagnosis and national treatment guidelines is crucial [10] and closely related to many socio-economic factors [11, 12], including the background of participants, their level of education, the type of health organization and years of experience [13].

In Equatorial Guinea, the National Programme for Malaria Control (NPMC) of the Ministry of Health and Social Welfare (MINSABS) updated its National Malaria Treatment Guidelines (NMTG) in 2008, establishing artesunate-amodiaquine (ASAQ) as first-line treatment for uncomplicated malaria. The use of oral artemisinin monotherapy was forbidden in Equatorial Guinea in 2014 [14]. Unfortunately, 9 years after the introduction of ASAQ therapy in Equatorial Guinea, compliance with the first-line treatment seems to be very poor in the Bata District [15].

Understanding local health workers’ treatment and diagnostic practices and knowledge is important in terms of improving malaria case management and disease control. This study sought to evaluate, for the first time, the practices and degree of knowledge regarding malaria and the National Malaria Treatment Guidelines amongst prescribers working at the public health facilities of Bata, located in the Continental Region of Equatorial Guinea.

## Methods

### Study design and setting

Equatorial Guinea (EG) is a sub-Saharan country located in the Gulf of Guinea. The Bata District, located on the coast of the Continental Region of Equatorial Guinea, is the largest district in the country, with a population of 309,345 inhabitants. Its capital is the city of Bata, one of the two main cities of EG, alongside Malabo, the country’s capital. The prevalence of malaria in children under 15 years of age is 41.2% [16].

The public health system structure of Bata District consists of 10 health centres, consisting of nine primary health care centres and one regional hospital, located in the city. Despite the state of public health institutions in the Bata District, patients have to pay for consultation, complementary tests and treatments. The management of these centres is heterogeneous and the prices, as well as the flow of patients and the availability of drugs, is different at each health facility. When data were collected for this study, only eight primary health care centres were operational (two rural centres and the rest urban).

The Equatorial Guinea NMTG recommends ASAQ as the first-line treatment for children > 2 months of age with malaria, whilst quinine (QN) is recommended for younger patients. Artemether-lumefantrine (AL) and QN remain second-line treatments if ASAQ is not effective. Intravenous artesunate is recommended for the management of severe malaria until the patient regains consciousness, then full ASAQ therapy should be administered. If artesunate is not available, quinine or artemether should be administered [17].

### Study population and data gathering

This cross-sectional survey was conducted in October-December 2017 amongst all public health professionals who attended patients under 15 years old with suspected malaria in the Bata District of Equatorial Guinea. Practitioners were asked about their practices and knowledge of malaria and the National Malaria Treatment Guidelines (NMTG). Questions about their sociodemographic characteristics, the biological cycle of malaria parasites, malaria treatment practices, the recommendations given by the NMTG and resistance to anti-malarial drugs were included in the questionnaire. Details of the survey have been described previously [15].

### Data analysis

A descriptive analysis of practitioners’ characteristics was carried out using frequency tables for categorical variables and mean and standard deviation or median and interquartile range for normally and not-normally distributed continuous variables, respectively. A bivariate analysis was carried out, with the dependent variable being the practitioner’s work centre: Health Centre or Regional Hospital. Differences in knowledge were assessed using the *χ*^2^ test. *P* values below 0.05 were considered significant.

In order to assess the effect of socio-demographic variables on practitioners' knowledge of malaria and the NMTG, a knowledge index was calculated, taking into account the answers they gave to some of the survey questions [18]. To achieve a maximum score of 13, the respondents had to know some general facts about malaria: that malaria is transmitted by a mosquito (1 point); symptoms of uncomplicated malaria such as fever, headache or weakness (1 point each); symptoms of severe malaria such as low level of consciousness, convulsions or respiratory depression (1 point each). Respondents also needed to know that diagnosis must be carried out using a microscope or a Rapid Diagnosis Test (1 point each). The survey also gauged whether they knew about different aspects of the NMTG, including: whether they were aware of the NMTG (1 point); the first line of treatment according to the NMTG in uncomplicated cases: ASAQ or Quinine (1 point); first-line treatment in complicated cases, such as intravenous (IV) AS, intravenous (IV) quinine or intramuscular (IM) artemether and oral treatment with ASAQ when possible (1 point). Practitioners also obtained 1 point if they knew about the existence of resistance to anti-malarial drugs. High and Low Knowledge of Malaria were defined as scores either above or within and below the overall median, respectively.

A logistic regression model was used to determine factors associated with a high knowledge of malaria score. The odds ratio (OR) and 95% confidence interval (95% CI) were computed; p-values less than or equal to 0.05 were considered statistically significant. Data analysis was performed using IBM SPSS statistics 23.

## Results

Out of a total of 44 practitioners who were interviewed, 59.1% worked at a Health Centre and 40.9% at the District Hospital of Bata (Table 1). Most of the practitioners at the hospital were women (83.3%), compared to 53.8% at the health centres. The median age of practitioners was 36 years (IQR: 31–44) and they had a median experience of 3 years (IQR: 2–7); the practitioners at the health centres were older and had more years of experience. All practitioners at the regional hospital were physicians, while only 38.5% at the health centres had a medical degree. At the regional hospital practitioners said to visit a significantly higher number of patients with malaria per day than practitioners at the health centres.

**Table 1:** Practitioners' demographic data and characteristics

	Health centre		Hospital		
	n	%	n	%	*P* value
Sex					
Male	12	46.15	3	16.67	
Female	14	53.85	15	83.33	0.042
Age					
20–29	1	3.85	3	16.67	
30–39	13	50.00	12	66.67	
40–49	5	19.23	1	5.56	
50–59	6	23.08	2	11.11	
> 60	1	3.85	0	0.00	0.246
Academic level					
Medical degree	10	38.46	18	100.00	
Nursing degree	5	19.23	0	0.00	
Nursing diploma	8	30.77	0	0.00	
Medical assistant	3	11.54	0	0.00	0.001
Years of experience					
< 5	17	65.38	15	83.33	
6–10	2	7.69	3	16.67	
11–20	3	11.54	0	0.00	
> 20	4	15.38	0	0.00	0.108
Malaria cases/day					
< 10	22	84.62	4	22.22	
11–20	4	15.38	9	50.00	
21–30	0	0.00	2	11.11	
> 30	0	0.00	3	16.67	0.000
< 15 years old malaria cases/day					
< 10	24	92.31	4	22.22	
11–20	2	7.69	9	50.00	
21–30	0	0.00	2	11.11	
> 30	0	0.00	3	16.67	0.000

### General knowledge regarding malaria

Regarding malaria transmission, 100% of practitioners knew that malaria is transmitted by a mosquito bite and 81.8% answered that the *Plasmodium* is the microorganism responsible for the disease, with no differences according to centre. Fever was mentioned by all practitioners, followed by vomiting (63.6%), headache (59.1%) and diarrhoea (50.0%). Anaemia and splenomegaly were mentioned by 13.6 and 11.4% of practitioners, respectively, when asked about uncomplicated malaria signs and symptoms. An additional table file shows this in more detail (Table 2).

**Table 2:** Knowledge about malaria, signs and symptoms

	Health centre		Hospital		
	n	%	n	%	*P* value
Malaria transmission					
Mosquito bite	26	100.00	18	100.00	–
Microorganism is responsible for the disease					
Plasmodium	22	84.62	14	77.78	
Other	4	15.38	4	22.22	0.563
Signs and symptoms of malaria					
Fever	26	100.00	18	100.00	–
Anaemia	5	19.23	1	5.56	0.194
Splenomegaly	4	15.38	1	5.56	0.312
Diarrhoea	15	57.69	7	38.89	0.220
Vomiting	15	57.69	13	72.22	0.325
Headache	16	61.54	10	55.56	0.691
Aching body	4	15.38	1	5.56	0.312
Chills	5	19.23	7	38.89	0.150
Loss of appetite	11	42.31	8	44.44	0.888
Vertigo	5	19.23	2	11.11	0.469
Sickness	2	7.69	0	0.00	0.228
Weakness	10	38.46	10	55.56	0.263
Abdominal pain	6	23.08	3	16.67	0.604
Signs and symptoms of severe malaria					
Loss of consciousness	4	15.38	0	0.00	0.081
Prostration	7	26.92	5	27.78	0.950
Convulsions	21	80.77	18	100.00	0.048
Acidotic breathing	1	3.85	0	0.00	0.400
Acute lung oedema	0	0.00	1	5.56	0.224
Circulatory collapse	1	3.85	1	5.56	0.789
Anaemia	14	53.84	7	38.88	0.329

Regarding the symptoms of severe malaria, convulsion was the symptom most mentioned (88.6%), being mentioned significantly more by practitioners at the regional hospital (*p* 0.048), followed by anaemia (47.7%) and prostration (27.3%), with no significant differences between practitioners at the health centres and the regional hospital.

### Knowledge and practices regarding diagnostic and treatment

Most of the practitioners said that malaria is diagnosed through clinical symptomatology, significantly more frequent at the health centres (Table 3). Microscopy confirmation for diagnosis was mentioned more frequently by practitioners at the regional hospital. Half of the health centre practitioners (50.0%) also stated that malaria could be diagnosed using Rapid Diagnostic Tests (RDTs), compared to only 16.67% of the hospital practitioners (*p* = 0.03). Practitioners were asked about which diagnostic method they preferred to use and microscopy, when available, was the most mentioned (59.1%), particularly by practitioners at the regional hospital (77.78%).

**Table 3:** Knowledge and practices regarding malaria diagnosis and treatment

	Health centre		Hospital		
	n	%	n	%	*P* value
Malaria diagnostic procedures					
Microscopy	25	96.15	18	100.00	0.400
Clinical symptomatology	25	96.15	14	77.78	0.059
RDT	13	50	3	16.67	0.024
Method you prefer to use					
Microscopy	12	46.15	14	77.78	
Clinical symptomatology	10	38.46	4	22.22	
RDT	4	15.38	0	0.00	0.066
Treatments you know for uncomplicated malaria in children under 15 years					
ASAQ	20	76.92	18	100.00	0.028
AL	19	73.08	17	94.44	0.071
QUININE	22	84.62	16	88.89	0.685
SP	14	53.85	9	50.00	0.802
AM im	21	80.77	15	83.33	0.828
AS oral	17	65.38	12	66.67	0.930
AS + SP	4	15.38	6	33.33	0.152
ASMF	3	11.54	3	16.67	0.626
Chloroquine	2	7.69	2	11.11	0.698
Treatment you prescribe to children under 15 years in cases of uncomplicated malaria					
ASAQ	5	19.23	5	27.78	
AL	5	19.23	12	66.67	
QUININE	1	3.85	0	0.00	
AS	2	7.69	1	5.56	
AM im	13	50	0	0.00	0.003
What makes you choose the treatment you mention?					
It is the most effective	17	65.38	12	66.67	0.93
It is the cheapest	2	7.69	3	16.67	0.356
Its availability	7	26.92	3	16.67	0.425
It is the safest	7	26.92	7	38.89	0.402
It is preferred by patients	5	19.23	1	5.56	0.194
It is indicated in the National Malaria Treatment Guidelines	3	11.54	1	5.56	0.497
Why not prescribe ASAQ?					
It is not available	5	19.23	2	11.11	0.469
Side-effects	14	53.85	12	66.67	0.395
It is not adapted to children	1	3.85	0	0.00	-
Patients do not like it	1	3.85	0	0.00	-
Others	4	15.38	2	11.11	0.439
Do you know if there are resistances to any anti-malarial drug in the Bata District?					
No	9	34.62	3	16.67	
Yes	17	65.38	15	83.33	0.166
Could you list which anti-malarial drugs have known resistances?					
Chloroquine	9	34.62	4	22.22	0.294
AS	1	3.85	5	27.78	0.034
AM	4	15.38	11	61.11	0.002
SP	2	7.69	1	5.56	0.638
ASAQ	3	11.54	1	5.56	0.455
Quinine	3	11.54	3	16.67	0.476

When talking about known treatments for uncomplicated malaria in children under 15 years old, 86.4% of the practitioners answered ASAQ, this being mentioned significantly more by practitioners at the regional hospital. Quinine was also mentioned by 86.4% of the respondents, but without any differences according to the place of work. The following treatments most mentioned were AL and intramuscular artemether (AM) (81.8%) with no significant differences according to place of work.

Concerning the most prescribed treatment for uncomplicated malaria in children less than 15 years old, significant differences emerged between treatment practices at the health centres and the regional hospital. Intramuscular artemether (AM) was the anti-malarial most mentioned by the practitioners at the health centres (50.0%), while the—artemether-lumefantrine (AL) was the treatment most mentioned by the practitioners at the regional hospital (66.7%). Furthermore, the practitioners who said to prescribe AM mentioned that they did so in association with sulfadoxine-pyrimethamine; this was mentioned significantly more frequently by those working at the health centres (53.8%) compared to the hospital (5.6%) (*p* = 0.001).

Regarding why they did not prescribe the first-line malaria treatment ASAQ, almost 60% of the practitioners mentioned its side-effects, without any differences according to work centres.

Concerning their knowledge of resistance to anti-malarial treatments in the Bata District, 72.3% of practitioners said to know about the existence of anti-malarial resistance. About 61.1% of the hospital practitioners mentioned AM, when asked about which drugs *P. falciparum* had developed a resistance to, compared to only 15.38% of the health centre practitioners (*p* value 0.002). Chloroquine was the second anti-malarial most mentioned by practitioners (29.5%), with no differences between work centres. Resistance to artemisinin was also mentioned significantly more by practitioners at the regional hospital (27.8%) than at the health centres (3.8%).

### The National Malaria Treatment Guidelines (NMTG)

Most of the practitioners at the health centres (57.7%) said not to know the National Malaria Treatment Guidelines (Table 4), while most practitioners working at the regional hospital said to know it (66.7%). Most of the practitioners who were aware of the Guidelines stated that they had heard about it through a colleague (39.1%), followed by specific MINSABS training (34.8%). Concerning the first-line treatment recommended in the NMTG for uncomplicated malaria in children less than 15 years old was, a 69.6% of practitioners mentioned ASAQ. However, only two practitioners answered correctly about the treatment protocol set out in the NMTG to treat a patient with severe malaria before transferring to the hospital, one from the hospital and one from a health centre, Regarding the treatment included in the NMTG for children less than 15 years old with severe malaria, 75.0% of practitioners working at the regional hospital answered correctly, while most of the practitioners working at the health centres cited other incorrect treatments. Most of the practitioners who knew the NMTG (65.2%) stated that they did not have any difficulty in applying the protocol (Table 4).

**Table 4:** National Malaria Treatment Guidelines (NMTG) knowledge and practices

	Health centre		Hospital		
	n	%	n	%	*P* value
Do you know the NMTG of Equatorial Guinea?					
No	15	57.69	6	33.33	
Yes	11	42.31	12	66.67	0.099
How did you hear about the NMTG?					
Specific MINSABS training	4	36.36	4	33.33	
Specific training of Centre’s Management	0	0.00	1	8.33	
Work colleagues	5	45.45	4	33.33	
Other training	2	18.18	3	25.00	0.736
First-line treatment regimen that NMTG recommends for children under 15 years					
ASAQ	7	63.64	9	75.00	
Quinine	0	0.00	2	16.67	
Other	4	36.36	1	8.33	0.134
First-line treatment guideline recommended by the NMTG before transferring					
AS suppositories	1	9.09	1	8.33	
No correct treatments	7	63.64	7	58.33	
Don´t know	3	27.27	4	33.33	0.100
First-line treatment regimen recommended by the NMTG for the treatment of severe malaria					
AS iv and, when tolerated, oral ASAQ	3	27.27	9	75.00	
Other	7	63.64	3	25.00	
Don´t know	1	9.09	0	0.00	0.062
Do you have any difficulty in applying the protocols proposed by the NMTG?					
No	8	72.73	7	58.33	
Yes	3	27.27	5	41.67	0.389
What are the main difficulties in applying the protocols in the NMTG?					
Presence of adverse effects	2	66.67	0	0.00	0.344
The patient does not like the treatment	0	0.00	2	40.00	0.162
Emergence of resistance	1	33.33	1	20.00	0.591
Lack of availability	0	0.00	2	40.00	0.162
Do you think that any change in the pattern of the NMTG protocol is necessary?					
No	8	72.73	10	83.33	
Yes	3	27.27	2	16.67	0.64

### Knowledge-related index and factors

**Fig. 1:**
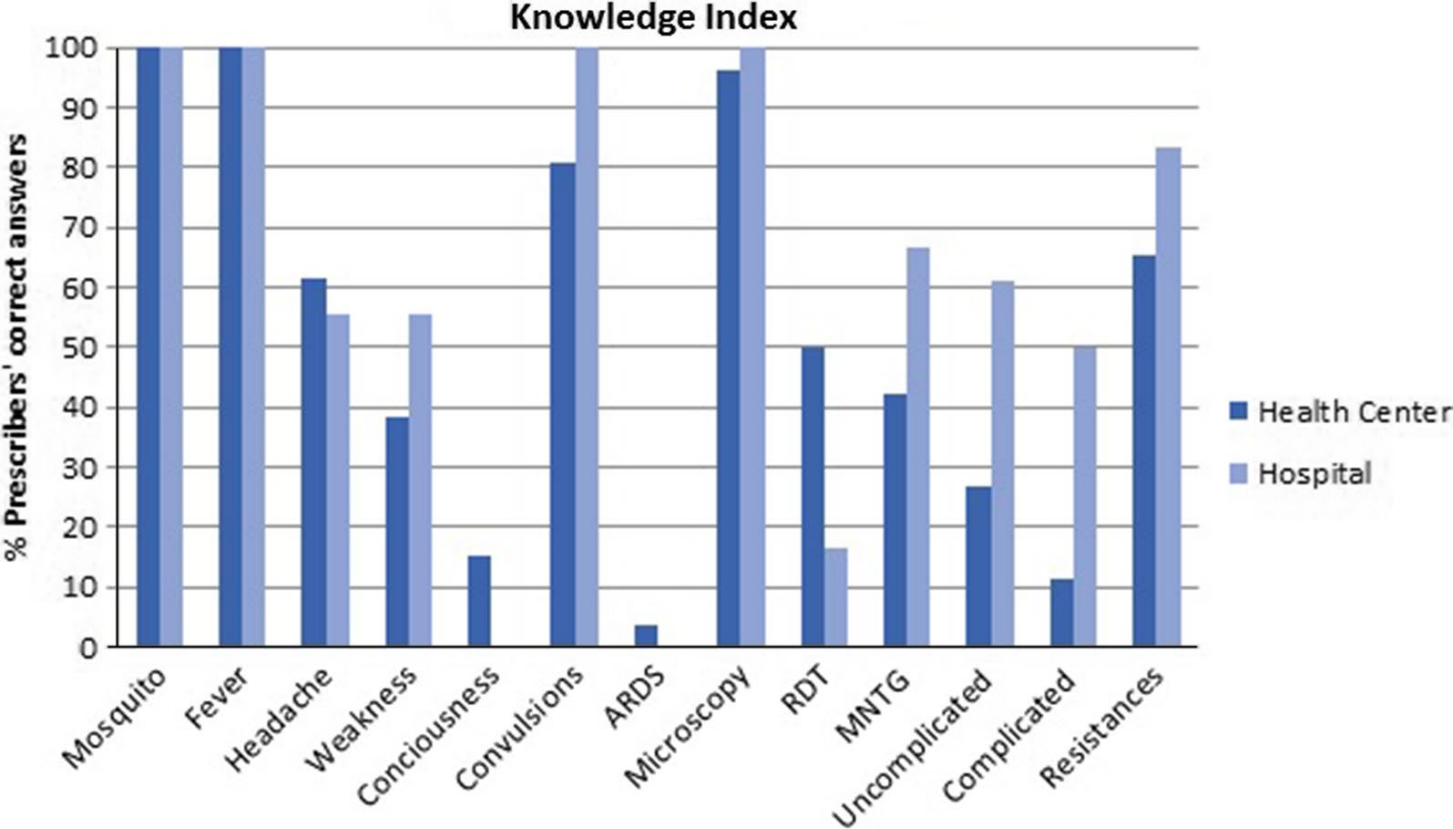
Knowledge index. Malaria and NMTG knowledge among public health prescribers in Bata District. (Mosquito): Malaria is transmitted by a mosquito bite. (Fever, Headache and Weakness): Fever, headache and weakness are symptoms of uncomplicated malaria. (Consciousness and Convulsions, ARDS): Loss of consciousness, convulsions and Acute Respiratory Distress Syndrome are symptoms of complicated malaria. (NTG): NMTG awareness. (Uncomplicated): NMTG’s first-line treatment for uncomplicated malaria. (Complicated): NMTG’s first-line treatment for complicated malaria. (Resistance): Anti-malarial resistance

The majority of Bata District practitioners (63.6%) had a low Malaria Knowledge Index, with none of the 44 practitioners interviewed achieving the maximum score of 13 points. The total median score was seven, with most practitioners from the health centres (76.92%) scoring Low and most of the practitioners from the regional hospital (61.11%) scoring above the median. Figure 1 shows this in more detail.

Following these results, a regression analysis was carried out to look for associations between the knowledge index of the practitioners and each of their sociodemographic characteristics. Prescribers with a medical degree, working at the hospital and visiting more than 11 malaria cases per day had a better knowledge of malaria and the NMTG, whilst years of experience were inversely associated with the level of knowledge (Table 5).

**Table 5:** Knowledge index and socio demographic characteristics

	Low	High	*P* value	OR	95% CI	
Sex						
Male	10	5				
Female	17	12	0.427	1.41	(0.38–5.20)	
Age						
< 35	11	10				
> 36	16	7	0.195	0.48	(0.14–1.65)	
Academic level						
Medical degree	14	2				
Others	13	15	0.007	8.08	(1.54–42.37)	
Years of experience						
< 3	12	13				
> 4	15	4	0.037	0.25	(0.06–0.10)	
Malaria cases/day						
< 12	19	7				
> 13	8	10	0.055	3.39	(0.95–12.09)	
< 15 years old malaria cases/day						
< 11	22	7				
> 12	5	10	0.008	6.29	(1.60–24.73)	
Health facility						
Health centre	20	6				
Hospital	7	11	0.013	5.24	(1.41–19.52)	

## Discussion

Adequate practices and knowledge about malaria and the NMTG are decisive in terms of ensuring proper management and the control of the disease [4]. However, low compliance with malaria national protocols by healthcare workers remains a problem in many countries in sub-Saharan Africa [19, 20]. Most practitioners working at public health facilities have a low level of knowledge regarding the National Malaria Treatment Guidelines (NMTG) in the Bata District of Equatorial Guinea. However, important differences between hospital and health centre workers were found. This study has found that better diagnostic, treatment practices, knowledge of malaria, the NMTG and treatment resistances was higher amongst hospital workers than amongst practitioners at health centres.

As in most African countries [3, 21], practitioners’ knowledge about malaria transmission, signs and symptoms was, in general, good. Although workers at the health centres mentioned more symptoms, convulsions related with severe malaria were mentioned significantly more by practitioners working at the regional hospital. Commonly, cases of severe malaria are more frequently seen at hospitals than at health centres [22].

Effective case management at public health facilities is one of the cornerstones of malaria control. Since 2012, the WHO has advised that clinical diagnoses should be confirmed by parasitological methods (6). In the Bata District, clinical diagnosis was the malaria diagnosis method most used by health centre workers, while confirmation by microscopy was mostly cited by those who worked at the regional hospital. Regarding RDTs, these were mentioned significantly more by the health centre workers Symptomatology continues to be more predominant for diagnosis than any laboratory test in most parts of Africa [23] and, frequently, practitioners request parasitology confirmation, but then pay little attention even to negative results, continuing to treat children as a malaria case [24].

Concerning knowledge and practices regarding malaria treatment, practitioners mentioned a wide range of different treatments for uncomplicated malaria in children under 15 years old, including treatments not recommended in the NMTG and former treatments, such as chloroquine. Concerning the treatment they prescribe most for uncomplicated malaria, intramuscular artemether was the therapy most mentioned by health centre workers, while AL was most frequently mentioned by workers at the regional hospital. Despite the fact that most of them knew that the first-line treatment in Equatorial Guinea is ASAQ, the side-effects were the main reason why they did not prescribe it. The same reasons and preference for AL compared to ASAQ has been described in many countries in the region [25, 26]. The use of injectable AM as a treatment for uncomplicated malaria has only been described in Sudan [27, 28] and Equatorial Guinea [15, 29]. This use of artemisinin monotherapy to treat uncomplicated malaria can contribute to the emergence of resistance to ACT and lead to treatment failure [30]. In the Bata District, public health practitioners knew about the existence of resistance to chloroquine, mostly mentioned by health centre practitioners, and resistance to AM mostly mentioned by hospital health workers, in accordance with the inadequate use of injectable AM by the practitioners at health centres [15].

The workers at the health centres said that they knew the National Malaria Treatment Guidelines, through a colleague. However, most of the practitioners working at the hospital knew the NMTG through specific MISABS training. The NMTG was introduced in the Continental Region of Equatorial Guinea in 2008, but, up until the time of this study, no reinforcement or follow-up training had been offered to malaria practitioners working at health centres regarding the national protocols for malaria management.

Despite this, ASAQ first-line treatment for children with uncomplicated malaria was well-known by those practitioners who knew the NMTG at both health facilities, although the first-line treatment for severe malaria was almost unknown to practitioners at the health centres. This could be also caused by the fact that this form of malaria is not meant to be treated at health centres, since it should be immediately referred to the hospital. Unfortunately, most of the practitioners at the health centres were also unaware of the malaria treatment protocol recommended before transferring the patient to the hospital [17]. Once again, knowledge regarding malaria case management must be improved in the Bata District of Equatorial Guinea through training if the aim is to achieve malaria control. Various initiatives implemented in other African countries could be taken into account [31].

The index of knowledge regarding malaria and the recommendations included in the NMTG of Equatorial Guinea is low amongst most health practitioners in the Bata District, being higher among hospital practitioners, who have fewer years of experience than the practitioners at the health centres, but who possess a higher degree of academic education. Low knowledge regarding malaria protocols is frequent in many African countries [32, 33], both in the private and public sector [8, 19, 34. 35]. Usually, the factors that influence local health workers’ knowledge of malaria frequently include education and working experience [33]. The comparison with regard to years of working experience reveals that practitioners with fewer years of working experience are more likely to have a solid knowledge of malaria than those with greater experience, due to the fact that they have received more recent and up-to-date training [13].

This study consisted of a cross-sectional survey of the Bata District, so the main limitation is that the results may not be generally applicable to the rest of the country, even when low compliance with first-line malaria treatment throughout the country is one of the main concerns of the National Programme for Malaria Control (NPMC).

## Conclusions

Practitioners at the Bata Regional Hospital had a higher knowledge of malaria and better practices than those working at the health centres. However, general knowledge about malaria and the National Malaria Treatment Guidelines is low in both groups. It is, therefore, essential to reinforce practitioners’ knowledge and use of the NMTG in order to improve malaria case management and disease control in the region.

## Data Availability

All data generated or analysed during this study are included in this published article.
